# Transmetatarsal Amputation Results in Higher Frequency of Revision Surgery and Higher Ambulation Rates Than Below-Knee Amputation

**DOI:** 10.1177/24730114221112938

**Published:** 2022-07-21

**Authors:** Angel Ordaz, Conner Trimm, Jason Pedowitz, Ian M. Foran

**Affiliations:** 1 University of California, San Diego, La Jolla, CA, USA

**Keywords:** transmetatarsal amputation, below-knee amputation, diabetes, foot ulcer

## Abstract

**Background:**

Selecting the level of amputation for patients with severe foot pathology can be challenging. The surgeon is sometimes confronted with an option between transmetatarsal amputation (TMA) and below-knee amputation (BKA). Recent studies have suggested that minor foot amputations have high revision rates and need for higher level of amputation. This study sought to compare the revision rates, need for higher level of amputation, postoperative ambulatory rate, and the demographic factors between these 2 operations.

**Methods:**

We retrospectively reviewed the records of patients undergoing either BKA or TMA at a single academic institution during an 8-year period. Demographic characteristics and medical history were collected and included in a binary logistic regression model to evaluate for independent predictors of needing revision surgery or needing higher-level amputation. Secondary outcomes included ambulatory status and wound status at last follow-up.

**Results:**

There was a total of 367 patients who underwent either BKA (n=293) or TMA (n=74).

On binary logistic regression, the only significant independent predictor of needing revision surgery was undergoing TMA (odds ratio [OR] 2.30, CI 1.199-4.146, *P* = .011). The presence of PAD trended toward significance (OR 2.12, CI 0.99-4.493, *P* = .051). Similarly, significant independent predictors of needing higher level amputation were undergoing TMA (OR 4.117, CI 1.9-8.9, *P* < .001) and presence of PAD (OR 4.85, CI 1.59-14.85, *P* = .006). More TMA patients were ambulatory (56.8%) on last follow-up compared with BKA patients (30.9%).

**Conclusion:**

Transmetatarsal amputation has a higher risk of reoperation and need for revision amputation compared with below-knee amputation. Transmetatarsal amputation has a higher chance of returning patients to independent ambulation. Patients with peripheral arterial disease are at a higher risk of revision surgery and higher-level amputation with both operations.

**Level of Evidence:**

Level III, retrospective case review.

## Introduction

Indicating the level for a lower extremity amputation can be challenging and is informed by multiple factors. One conundrum that sometimes arises is whether to perform a transmetatarsal amputation (TMA) or a below-knee amputation (BKA). In some cases, the decision is dictated squarely by local soft tissue trauma or infection (eg, a patient with calcaneus osteomyelitis or a mangled foot is not a candidate for a TMA). However, in many cases, the local factors may leave the surgeon and patient with the option of both operations.

Transmetatarsal amputation may seem like the obvious choice as it a faster and technically simpler operation, results in less blood loss, preserves lower extremity length thereby improving energy conservation, and is cosmetically desirable for patients.^2-4,8,9,11,17^ Successful transmetatarsal amputation also leads to better rates of ambulation than BKA.^1,4,9^ However, one downside is that TMA may theoretically lead to a higher frequency of revision surgery, including higher level of amputation compared with BKA.^9,11^ Several studies have shown a high rate of need for reoperations including irrigation and debridement and revision amputation with foot amputations.^3-14,15,20^ Prolonged wound healing or failure of TMA with resultant need for revision surgery can result in prolonged weightbearing restrictions, prolonged need for wound care and antibiotics, hospital readmission, multiple anesthetic episodes, and generally significant morbidity for patients who are often already deconditioned and have multiple medical comorbidities.^4,11^ Failure of TMA also results in a considerable resource and financial cost burden for the health care system.^9^

Acknowledging that there may be confounders related to medical comorbidities and patient selection, the primary aim of this retrospective case series was to compare the need for higher level of amputation in TMA vs BKA. We also looked at several other common indicators of success after lower extremity amputation between the 2 groups. Better understanding of the differential complications between these 2 different procedures may help surgeons better indicate and counsel patients for these procedures.

## Methods

After approval of our Institutional Review Board, a retrospective analysis was performed on patients who had undergone either BKA or TMA by vascular or orthopaedic surgeons at a single academic center between January 2013 and May 2021. Attending (supervising) surgeons were present and performed all vital portions of the cases and were fellowship trained in either orthopaedic trauma, orthopaedic foot and ankle, or vascular surgery. Patient information from electronic health records was archived in the Health Insurance Portability and Accountability Act–compliant online Research Electronic Data Capture (REDCap) database (Vanderbilt University). Patients were excluded if they were below the age of 16 years.

Demographic data collected included patient age, gender, body mass index (BMI), history of smoking, and presence or absence of diabetes, coronary artery disease (CAD), peripheral arterial disease (PAD), chronic kidney disease (CKD), and Charcot neuroarthropathy. The presence or absence of PAD was determined by a combination of chart history, transcutaneous oximetry, and doppler vascular examinations, although not all patients had each of these components in their chart. History of previous revascularizations in the ipsilateral extremity undergoing amputation was determined. Patient laboratory values were collected, including hemoglobin A_1c_, creatinine, and glomerular filtration rate (GFR). Surgeon specialty performing the operation (orthopaedic vs vascular) was determined. Primary outcome variables were revision surgery and if patients underwent higher level of amputation. These outcome variables were compared between orthopaedic and vascular services. Other outcomes collected included hospital length of stay (HLOS), follow-up time ambulatory status at last follow-up, wound status at last follow-up, and reasons for revision surgery.

Descriptive variables are presented as either frequencies or mean values with SDs. Categorical variables were compared between patients undergoing either TMA or BKA with a χ^2^ test. Continuous variables were compared using an independent *t* test. Binary logistic regression was used to determine differences in the need for revision surgery or higher-level amputation between TMA and BKA groups. Any significantly different demographics or characteristics were added into the regression model to account for potential confounders. Statistical significance was defined as *P* <.05. All statistical analyses were performed using SPSS, version 28.00 (IBM Corp, Armonk, NY).

## Results

### Demographics and Characteristics

A total of 367 patients were included in the analysis. There were 293 patients in the BKA group and 74 patients in the TMA group. On average, BKA patients were younger (57.3 ± 13.2 years) than TMA patients (61.5 ± 11 years). There were 223 men (76.1%) in the BKA group and 54 men (73%) in the TMA group. Mean follow-up time was 315.1 ± 531.8 days in the BKA group and 181 ± 310.4 days in the TMA group. Mean BMI was 27.3 ± 6.8 and 27.4 ± 6.4 in the BKA and TMA groups, respectively. Mean creatinine, GFR, and HbA_1c_ laboratory values in the BKA group were 1.9 ± 2, 47.9 ± 19, and 8.2 ± 2.7, respectively. In the TMA group, these laboratory values were 2.5 ± 2.6, 42.7 ± 21.8, and 7.4 ± 2.1, respectively. Average HbA_1c_ was significantly higher in the BKA group. Prevalence of diabetes, CAD, PAD, CKD, and Charcot neuroarthropathy were, respectively, 69.3%, 24.1%, 38.9%, 37.1%, and 11.3% in the BKA group and 82.4%, 73%, 60.8%, 51.4%, and 0% in the TMA group. The prevalence of PAD and diabetes was significantly higher in the TMA group. The prevalence of Charcot neuroarthropathy was significantly higher in the BKA group. There were significantly more previous revascularizations in TMA patients (47.2%) than in BKA patients (22.8%). There were significantly more smokers in the BKA group (23.3%) than in the TMA group (11%). The orthopaedic surgery service performed 57.3% of BKA procedures and 31.1% of TMA procedures. These findings are detailed in Table 1.

**Table 1.** Demographic and Clinical Characteristics of Patients Undergoing BKA vs TMA.

Descriptive Variable	BKA (n=293)	TMA (n=74)	***P*** Value
**Medical history**			
Age, y, mean (SD)	57.3 (13.2)	61.5 (11)	.013*
BMI	27.3 (6.8)	27.4 (6.4)	.910
History of CAD, %	24.1	27	.651
History of Charcot joint, %	11.3	0	.002*
History of CKD, %	37.1	51.4	.031*
History of diabetes, %	69.3	82.4	.029*
Male, %	76.1	73	.191
PAD, %	38.9	60.8	<.001*
Smoking history, %	23.3	11	.023*
Previous revascularization, %	22.8	47.2	<.001*
**Laboratory values, mean (SD)**			
Hemoglobin A_**1c**_	8.2 (2.7)	7.4 (2.1)	.017*
Creatinine, mg/dL	1.9 (2)	2.5 (2.6)	.061
GFR, mL/min/1.73 m^2^	47.9 (19)	42.7 (21.8)	.068
**Service**			
Orthopaedic surgery, %	57.3	31.1	<.001
Vascular surgery, %	42.7	68.9	<.001

Abbreviations: BKA, below-knee amputation; BMI, body mass index; CAD, coronary artery disease; CKD, chronic kidney disease; GFR, glomerular filtration rate; PAD, peripheral artery disease; TMA, transmetatarsal amputation.

* Statistically significant at *P* < .05.

### Outcomes and Binary Logistic Regression

On univariate analysis, the TMA group had a significantly higher percentage of patients who required revision surgery (48.6%) and higher-level amputation (31.5%) when compared to the BKA group (23.2% and 9%, respectively) (*P* < .001). HLOS was significantly longer in the BKA group (17.3 ± 21.2 days) than in the TMA group (13.6 ± 10.9 days) (*P* = .04). A significantly higher percentage of TMA patients were ambulatory (56.8%) on last follow-up when compared to BKA patients (30.9%) (*P* < .001). The percentage of patients with fully healed amputation sites at last follow-up was not significantly different between BKA (58.3%) and TMA (51.4%) groups (*P* = .295). These findings, along with reasons for revision surgery or higher-level amputation in each group, are listed in Table 2.

**Table 2.** Univariate Analysis of Clinical Outcomes in Patients Undergoing Amputation BKA or TMA.

Outcome Variable	BKA (n=293)	TMA (n=74)	***P*** Value
Revision surgery, %	23.2	48.6	<.001*
Higher-level amputation, %	9	31.5	<.001*
HLOS, d, mean (SD)	17.3 (21.2)	13.6 (10.9)	.04*
Ambulatory at last F/U, %	30.9	56.8	<.001*
Wound at last F/U, %	58.3	51.4	.295
**Reason for revision surgery/higher level amputation**, %			
Wound breakdown	11.9	27.1	.2
Infection	15	33.8	<.001*
Gangrene	2	5.4	.121
Other	4.1	14.9	<.001

Abbreviations: BKA, below-knee amputation; F/U, follow-up; HLOS, hospital length of stay; TMA, transmetatarsal amputation.

* Statistically significant at *P* <.05.

Primary outcomes were broken down by which service performed the amputation (vascular vs orthopaedics). When looking at a univariate comparison between services, patients undergoing an amputation by the vascular service had a higher percentage of requiring revision surgery (39.2%) than when performed by the orthopaedic service (18.3%) (*P* < .001). Similarly, vascular surgery patients had a higher percentage of requiring a higher-level amputation (21.5%), when compared to orthopaedic surgery patients (6.3%) (*P* < .001). These outcomes are further broken down by whether patients were undergoing either BKA or TMA, with details listed in Table 3.

**Table 3.** Univariate Analysis of Outcomes Orthopaedic and Vascular Services.

Outcome	Vascular, %	Orthopaedic, %	***P*** Value
**Revision surgery**	39.2	18.3	<.001*
BKAs requiring revision	32	16.7	.002*
TMAs requiring revision	56.9	30.4	.039*
Higher Level Amputation	21.5	6.3	<.001*
BKAs requiring higher-level amputation	14.8	4.8	.003*
TMAs requiring higher-level amputation	38	17.4	.078*

Abbreviations: BKA, below-knee amputation; TMA, transmetatarsal amputation.

* Statistically significant at *P* < .05.

On binary logistic regression, the only significant independent predictor of needing revision surgery was undergoing TMA (odds ratio [OR] 2.30, CI 1.199-4.146, *P* = .011). The presence of PAD trended toward significance (OR 2.12, CI 0.99-4.493, *P* = .051). Similarly, significant independent predictors of needing higher-level amputation were undergoing TMA (OR 4.117, CI 1.9-8.9, *P* < .001) and presence of PAD (OR 4.85, CI 1.59-14.85, *P* = .006). Although orthopaedic and vascular services had significantly different percentages of requiring revision surgery or higher-level amputations, regardless of type of amputation, surgical service did not predict these outcomes on multivariate analysis as seen in the last rows of Tables 4 and 5.

**Table 4.** Multivariable Predictors for Need for Revision Surgery.^a^

Predictor Variable	OR (95% CI)	***P*** Value
TMA vs BKA	2.230 (1.199, 4.146)	.011*
Age	0.991 (0.966, 1.017)	.488
Hemoglobin A_**1c**_	0.981 (0.863, 1.115)	.773
History of diabetes	0.822 (0.398, 1.697)	.596
History of PAD	2.115 (0.996, 4.493)	.051
History of CKD	1.147 (0.638, 2.062)	.648
History of smoking	0.710 (0.350, 1.438)	.342
Charcot joint	0.314 (0.089, 1.116)	.074
Previous revascularizations	1.241 (0.614, 2.509)	.548
Orthopaedic performed procedure	0.822 (0.407, 1.658)	.584

Abbreviations: BKA, below-knee amputation; CKD, chronic kidney disease; OR, odds ratio; PAD, peripheral artery disease; TMA, transmetatarsal amputation.

a Multivariable logistic regression for each predictor variable was significantly different between groups on univariate analysis.

* Statistically significant at *P* <.05.

**Table 5.** Multivariable Predictors for Need for Higher Level of Amputation.^a^

Predictor Variable	OR (95% CI)	***P*** Value
TMA vs BKA	4.117 (1.906, 8.895)	<.001*
Age	1.006 (0.961, 1.042)	.745
Hemoglobin A_**1**c_	1.069 (0.878, 1.302)	.506
History of diabetes	1.182 (0.448, 3.124)	.735
History of PAD	4.854 (1.587, 14.847)	.006*
History of CKD	0.839 (0.377, 1.866)	.667
History of smoking	0.419 (0.162, 1.079)	.071
Previous revascularizations	0.656 (0.271, 1.595)	.395
Orthopaedic performed procedure	0.585 (0.215, 1.595)	.295

Abbreviations: BKA, below-knee amputation; CKD, chronic kidney disease; OR, odds ratio; PAD, peripheral artery disease; TMA, transmetatarsal amputation.

a Multivariable logistic regression for each predictor includes baseline comorbidities and history of prior ipsilateral trans metatarsal amputations or revascularization procedures. Predictors with *P* <.10 on logistic regression are shown.

* Statistically significant at *P* <.05.

## Discussion

Selecting the level of amputation for patients with severe foot pathology such as osteomyelitis or gangrene can be challenging. When faced with the choice between TMA and BKA, patients generally prefer to maintain as much of their limb as possible. Surgeons, likewise, may prefer TMA with the hopes of improving patients’ long-term energy expenditure, ambulation status, and cosmesis. However, mounting evidence suggest that well-meaning attempts at foot salvage can result in greater overall long-term morbidity.^3-15,20^ Foot amputations such as transmetatarsal, Chopart, and Lisfranc amputations are associated with high reoperation rates and need for revision surgery.

The primary finding of our study was that TMA results in a higher rate of revision amputation and a higher rate of any reoperation than BKA even when controlling for confounding variables. A recent study that queried the NSQIP database found that 30-day reoperation for irrigation and debridement (I&D) was about 2 times higher in minor foot amputations (Chopart, Lisfranc, transmetatarsal) compared with BKA.^9^ This is in keeping with our own findings, where TMA had an OR of about 2 compared with BKA for reoperation and an OR of about 4 compared with BKA for revision amputation. Our study’s revision amputation and reoperation rates for TMA and BKA are in keeping with other studies, without limiting follow-up to within 30 days.

Our study found that the return to independent ambulation after TMA was about 25% higher than BKA (57% vs 31%). The poor rate of return to independent or previous level of ambulation after BKA is well established and is thought to be particularly low in older patients.^9^ This highlights a major potential benefit of TMA that has been echoed by others.^4,9,17^ Independent ambulation likely improves both long-term morbidity and mortality and quality of life.

Multiple studies have attempted to identify risk factors for failure of partial foot amputations to help improve indications for these procedures.^1,3,5,7,10,11,13,15,18-20^ Identified risk factors vary between studies, but have included PAD,^2,7,13,17^ neuropathy,^7^ CKD,^16^ and revascularization performed around the time of the index amputation.^18,19^ The only independent comorbidity identified as an independent predictor of need for revision amputation or reoperation in our study was PAD. Interestingly, PAD was more common in the TMA group, although TMA remained an independent predictor of requiring revision surgery or higher-level amputation even with PAD being added to the logistic regression model. There is controversy regarding whether revascularization prior to amputation can improve outcomes, but in patients with severe PAD and absent pulses, consultation with a vascular surgeon is certainly warranted.^18,19^

Although univariate analysis demonstrated that patients undergoing amputation (BKA or TMA) with vascular surgery had a higher percentage of requiring revision surgery and higher-level amputations, this was most likely due to the higher number of patients with PAD in the vascular surgery group, and the higher percentage of TMA patients vascular surgeons managed. On multivariate logistic regression, PAD and TMA remained the only notable independent predictors of requiring a higher-level amputation or revision surgery, and whether orthopaedics or vascular performed the procedure did not matter in these analyses (Tables 4 and 5).

Overall, our study validates the notion that although TMA has a superior functional operation compared to BKA, it has a higher chance of resulting in reoperation or higher level of amputation. Patients with peripheral arterial disease are at a particularly high risk of failure, regardless of whether undergoing BKA or TMA. In a separate analysis not shown, when analyzing only patients with PAD, TMA patients were even more likely to require revision surgery and higher-level amputations. This is unsurprising given that both TMA and PAD were independent risk factors for these outcomes in the binary logistic regression model. Although these findings are unlikely to dissuade patients for attempting TMA, it can help to temper expectations and mentally prepare them when TMA fails.

It is important to note that revision surgery/amputation and ambulatory status are not the only considerations when making a decision between these 2 procedures. Our study did not analyze perioperative mortality. Several studies have shown that BKA has a high perioperative mortality rate.^2,9,12^ Below-knee amputation has also been shown to have a higher rate of transfusion, and our study showed a longer hospital length of stay.^9^ These factors should also be considered when indicating a patient for surgery.

Our study has limitations. It is a retrospective review of a heterogenous group of patients who underwent lower extremity amputation. The retrospective nature of this study makes chart review vulnerable to misclassification bias. The patients in the different groups were not matched. Patients in the 2 groups had different rates of medical comorbidities, although we attempted to control for this with multivariate regression analysis. The multivariate regression model did not consider all possible confounders leading to poor outcomes, including variables such as operative time, prior foot deformity, adjunct procedures, and wound location. Indication to perform TMA or BKA was made at the discretion of the attending surgeon. Finally, there were a greater number of BKA patients than TMA patients, given the overall lower frequency of TMAs performed at this institution, although we recognize this can bias the study results.

## Conclusion

Transmetatarsal amputation has a higher risk of reoperation and need for revision amputation compared to BKA, but TMA has a higher chance of returning patients to independent ambulation. Patients with peripheral arterial disease are at a higher risk of revision surgery and higher-level amputation whether BKA or TMA is performed.
